# Dynamic changes of Receptor activator of nuclear factor-κB expression in Circulating Tumor Cells during Denosumab predict treatment effectiveness in Metastatic Breast Cancer

**DOI:** 10.1038/s41598-020-58339-2

**Published:** 2020-01-28

**Authors:** Francesco Pantano, Elisabetta Rossi, Michele Iuliani, Antonella Facchinetti, Sonia Simonetti, Giulia Ribelli, Alice Zoccoli, Bruno Vincenzi, Giuseppe Tonini, Rita Zamarchi, Daniele Santini

**Affiliations:** 1 Medical Oncology Department of Campus Bio-Medico, University of Rome, Rome, Italy; 2 Department of Surgery, Oncology and Gastroenterology, University of Padova, Padova, Italy; 3 Veneto Institute of Oncology IOV-IRCCS, Padua, Italy

**Keywords:** Bone metastases, Breast cancer

## Abstract

Receptor-activator of nuclear-factor –κB-ligand (RANKL) and its receptor RANK have been recently identified as key players in breast cancer bone metastases. Since Circulating Tumor Cells (CTCs) are considered a crucial step of metastatic process, we explored RANK expression on CTCs in metastatic breast cancer (MBC), and the predictive value of RANK-positive CTCs in monitoring patients during treatment with denosumab (anti-RANKL antibody). To this purpose, we developed a novel CTC assay to quantify RANK-positive CTCs in forty-two bone MBC patients, candidates to denosumab treatment. Companion algorithms ΔAUC and Slope were developed, and correlated with time to first skeletal-related-events (SRE), time to bone metastasis progression and time to visceral metastasis progression. Twenty-seven patients had at least one CTC at baseline and, among these, nineteen (70%) had one or more RANK–positive CTCs. Notably, the baseline total CTCs, but not the RANK-positive, were associated with Time-to-first-SRE, Time-to-Bone-Metastasis-Progression and Time-to-Visceral-Metastasis-Progression. Conversely, during treatment monitoring, positive ΔAUC value, expression of RANK-positive CTCs persistence, correlated with longer Time-to-first-SRE (p = 0.0002) and Time-to-Bone-Metastasis-Progression (p = 0.0012). Furthermore, the early increase at second day, in RANK-positive CTCs (Positive-Slope) was associated with delay in time-to-first-SRE (p = 0.0038) and Time-to-Bone-Metastasis-Progression (p = 0.0024). We demonstrate, for the first time, the expression of RANK on CTCs in MBC patients and that the persistence of RANK expression determines denosumab effectiveness.

## Introduction

Receptor activator of nuclear factor-kappaB ligand (RANKL)/RANK axis represents the main characterized therapeutic target to prevent bone metastasis in solid tumors^1^. About 70% metastatic breast cancer (MBC) patients develops skeletal metastases often associated to skeletal related events (SREs) with a serious negative impact on patients’ quality of life^2^.

Accumulating evidences suggest that RANKL/RANK signaling, not only influences bone microenvironment, mediating osteoclast activity and survival, but also promotes BC metastases^1,3^. Indeed, RANK-RANKL blocking antibodies showed antitumor efficacy in bone MBC preclinical models both in preventive^4,5^ and therapeutic settings^4,6,7^. This antitumor effect could be either “indirect” through the inhibition of bone resorption that, in turn, prevents the development of skeletal metastasis or “direct” blocking RANK-expressing tumor cells invasion and migration. Several studies showed that RANK expression on tumor cells induced stemness and epithelial mesenchymal transition promoting tumor progression, and metastasis^8–16^. In this regard, our group has previously demonstrated that RANK expression on primary BC was associated with poor prognosis in terms of bone metastasis relapse^17,18^.

Denosumab, a monoclonal human antibody against RANKL, represents the gold standard for preventing SREs onset in bone metastatic patients. Data from clinical trials suggested that denosumab treatment also improved Overall Survival (OS) in metastatic patients. In particular, a survival advantage was found in bone metastatic lung cancer patients treated with denosumab compared to zoledronic acid^19^; similarly, denosumab delayed the time of bone metastasis onset in metastatic prostate cancer^20^. Moreover, in adjuvant setting (ABCSG-18 trial), non-metastatic BC patients receiving denosumab in association with adjuvant aromatase inhibitor, showed a longer disease-free survival compared to those treated with aromatase inhibitor and placebo^21^. Conversely, data from D-CARE trial showed that adjuvant denosumab does not reduce tumor relapse or deaths in high-risk early BC patients treated with standard loco-regional and neoadjuvant therapy^22^. Hence, the direct antitumor effect of denosumab is still on debate and need to further investigations in clinical and translational setting.

Tumor cells - single or as tumor micro-emboli - circulate in the peripheral blood of cancer patients and might colonize secondary site, producing metastasis^23^. Named CTCs, i.e. circulating tumor cells, these cells have been investigated as potential predictive biomarker of treatment efficacy, and correlated with PFS in several tumors, as BC^24^.

In addition, CTC detection has been regarded as a promising early diagnostic marker and prognostic tool useful to monitor disease progression and select the optimal treatment strategies. In several studies, high CTC count has been correlated to a worse outcome^25,26^, whilst a reduction in CTC count matched with treatment efficacy^26,27^.

Different methodologies for CTC detection and analysis have been developed, but CellSearch (CS) is the unique platform Food and Drug Administration approved to enumerate CTCs in MBC. This approval was obtained following the encouraging results reported by *Cristofanilli et al*., that demonstrated a significant correlation between CTC count, by CS, and clinical outcome in MBC patients^25^. In particular, MBC patients with ≥5 CTCs per 7.5 mL of peripheral blood had a reduced PFS and OS^25^. Ten years later, the independent prognostic value of baseline CTC enumeration was confirmed by a European pooled analysis on 1944 MBC patients^26,27^. Finally, a recent international expert consensus paper has been proposing the cut-off value of 5 or more CTCs as a prognostic tool for stratifying MBC patients in Stage IV_*indolent*_ and Stage IV_*aggressive*_ subgroups^28^.

However, along with CS, in these years, several open platforms have been proposed, to better enrich and characterize different subsets of CTCs, especially those in epithelial-to-mesenchymal-transition whom role is crucial in metastatic process. To date, none procedure reached the demonstration of clinical validity of Level 1 of evidence as the CS^27^, that is still the gold-standard for enrich and quantify CTCs^29^.

CTCs are heterogeneous cells that could dynamically change their molecular profile during cancer treatment^30–34^. Hence, addressing the role and mechanisms of RANKL/RANK axis in metastatic process, we planned to explore whether RANK is expressed on cellular membrane of CTCs in MBC patients, as primary objective of our pilot study.

To this purpose, we developed a novel CTC assay by using an anti-RANK mAb in conjunction with CS platform, since it permits serial testing with good sensitivity and reproducibility. We then investigated if the analysis of RANK-positive CTCs could have a predictive value in monitoring MBC patients’ outcomes during denosumab treatment (secondary objective).

## Results

### RANK positive CTC were detectable in the majority of MBC patients

From 2012 to 2015, we examined 42 consecutive MBC patients with skeletal metastases candidate to denosumab therapy. Table 1 summarizes patients’ characteristics.

**Table 1:** Demographics and clinical parameters.

Clinical parameters	Patients (N = 42)
**Age at baseline (y/o)**	
Median	61
Range	32–89
<55	18 (43%)
≥55	24 (57%)
**Menopausal status**	
Premenopausal	11 (26%)
Postmenopausal	31 (74%)
**Tumor intrinsic subtype**	
ER + HER2- Ki-67 < 15%	15 (36%)
ER + HER2- Ki-67 ≥ 15%	15 (36%)
HER2 amplified	9 (21%)
Triple negative	3 (7%)
**Concomitant oncological treatment**	
Hormone therapy	22 (52%)
Chemotherapy only	13 (31%)
Anti-HER2 agents + hormone therapy	3 (7%)
Chemotherapy + anti-HER2 agents	4 (10%)
**Visceral metastasis at baseline**	
Absent	23 (55%)
Present	19 (45%)
**Type of bone metastasis**	
Lytic	14 (33%)
Sclerotic	15 (36%)
Mixed	13 (31%)

Median age was 61 years (range, 32–89). The histological subtypes included ER + HER2- Ki-67 < 15% (15 cases, 36%), ER + HER2- Ki-67 ≥ 15% (15 cases, 36%), triple-negative (3 cases, 7%), and HER2 amplified (9 cases, 21%). All patients included in the study had an ECOG performance status 0.

At enrollment, 21 out of 42 patients were recurrent breast cancer, and 21 were de novo MBC. All recurrent patients had been treated with adjuvant treatment according to the clinical-pathological factors; whilst de novo MBC did not received any prior systemic treatment.

As previously reported for another customized assay^35^, we exploited the novel test to enumerate both total and RANK-positive CTCs in the same sample. Twenty-seven (64%) out of 42 MBC patients had at least one CTC at baseline, 12 (29%) patients had five or more CTCs. Furthermore, among the 27 CTC-positive patients at baseline, 19 (70%) had one or more RANK–positive CTCs. The baseline number of CTCs and RANK-positive CTCs ranged from 0 to 140 (median 2) and 0 to 7 (median 0) cells, respectively.

**Figure 1:**
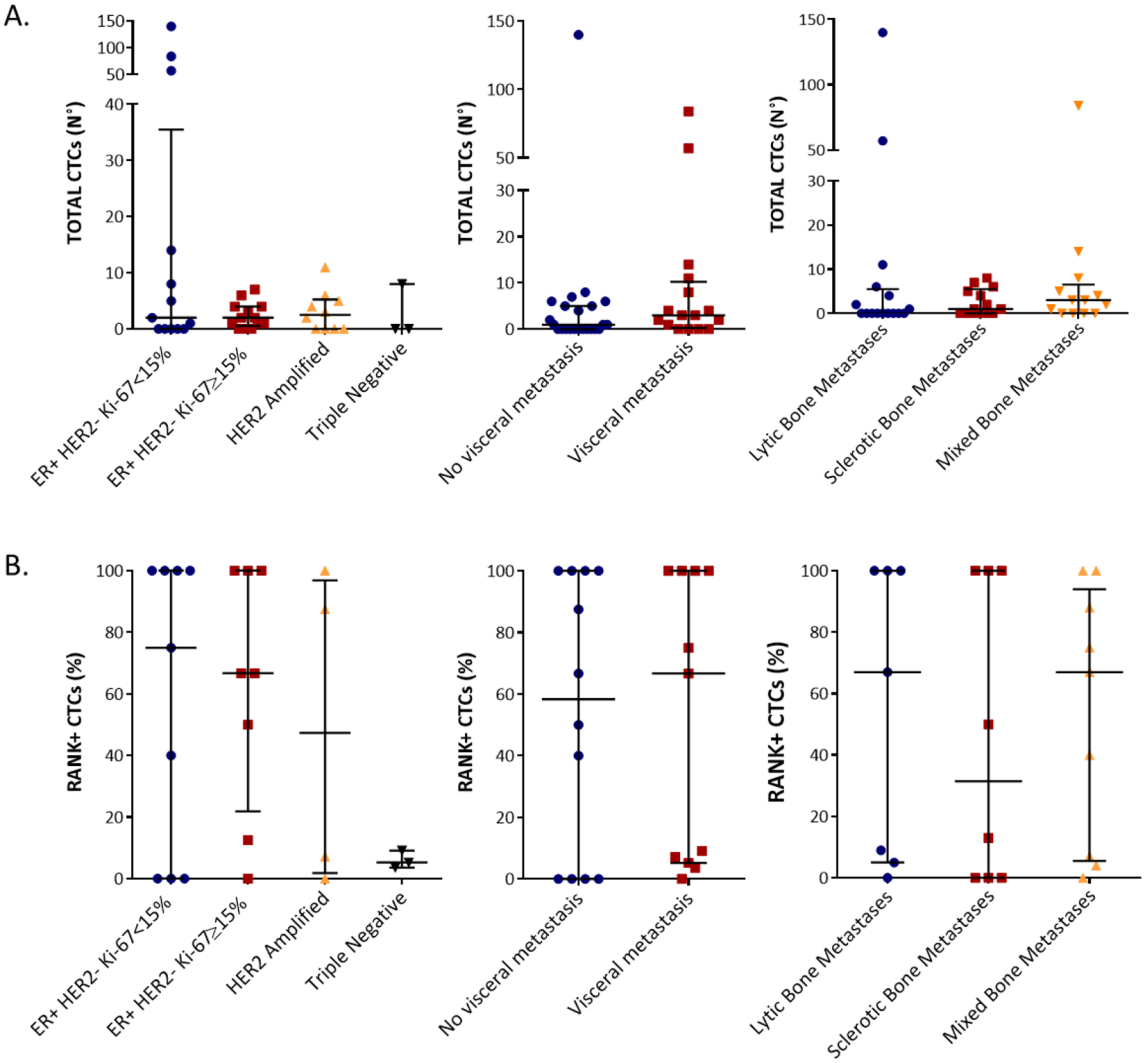
CTCs count according to tumor subtypes and metastasis presence. Total CTCs count (**A**) and RANK positive CTCs percentage (**B**) related to different breast tumor intrinsic subtypes, presence of visceral metastasis and bone metastasis type.

Neither total CTC nor RANK-positive CTC enumeration at baseline was associated with any specific tumor intrinsic subtype, presence of visceral metastases or bone metastasis type. (Fig. 1).

### Association between CTC subsets and clinical parameters or outcome

We then investigated whether RANK-positive CTCs and their changes during treatment may predict therapeutic response, in terms of time to first SRE, time to bone and visceral metastasis progression.

To this purpose, we collected peripheral blood samples to assess total and RANK-positive CTCs at different time points, namely baseline and at day 2, 7, 14, 28 (T0, T1, T2, T3, and T4) after the first denosumab administration. The sample size in the timeline schedule was of 39, 34, 34, 30 and 34 evaluable samples, for T0, T1, T2, T3, and T4, respectively.

The choice of the timeline schedule was based on *in vitro* data^36^, showing that short-term culture (2 days) can detect functional effects of RANKL variants on osteoclastogenic capacity, and on *in vivo* studies of pharmacokinetics and pharmacodynamics of denosumab^37^. Indeed, in advanced cancer patients with bone metastases, denosumab reach a peak in serum within the first week, and bone resorption decreases significantly as early as 1 day after administration of denosumab^37^.

Moreover, we extended the observation time weekly up to the 4^th^ week, in order to include a time-point usually exploited in CTC studies to evaluate early changes of CTC level, which were reported to improve prognostic accuracy of baseline CTC test^25,27^. As expected^27^, univariate analysis showed that total CTC count at T0 was significant associated with higher risk of reduced time to first SRE development and bone and visceral metastasis progression; on the contrary RANK-positive CTC count at T0 did not correlate with clinical endpoints (Table 2).

**Table 2:** Univariate Cox regression analysis of Total CTCs and RANK + CTCs at T0.

Variables	Time to first SRE		Time to bone metastasis progression		Time to visceral metastasis progression	
	p value	HR (95% CI)	p value	HR (95% CI)	p value	HR (95% CI)
Total CTCs T0	0.002	1.02 (1.01–1.03)	0.001	1.02 (1.01–1.03)	0.001	1.02 (1.01–1.03)
RANK + CTCs T0	0.689	1.05 (0.84–1.31)	0.842	0.98 (0.79–1.22)	0.681	1.04 (0.86–1.25)

Abbreviation = SREs, Skeletal Related Events.

**Figure 2:**
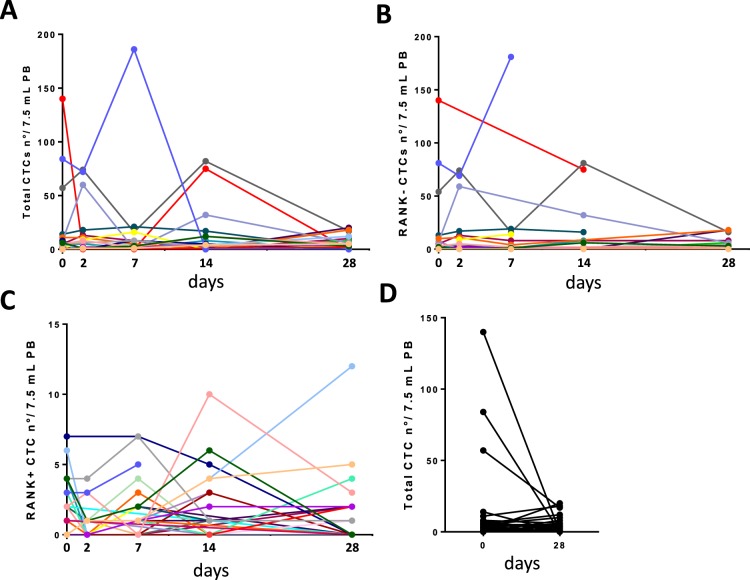
CTCs counts from baseline to the 4^th^ week. Line charts of individual serial counts of total (**A**), RANK-negative (**B**) and RANK-positive CTCs (**C**) from T0 to T4. The levels of total CTCs at baseline (T0) and at the end of the first cycle of denosumab (T4) have been also show in a separate plot (**D**).

Concerning the value of monitoring a subset of CTCs in relation to time-schedule of a specific treatment, the Fig. 2 shows the changes in total, RANK-negative and RANK-positive CTCs over the follow-up period. As previously reported in other studies^35^, the individual longitudinal graphs of CTCs showed a growing and waning profile that seems arduous to interpret and to manage for clinical purpose. Moreover, although the total CTCs number detected at T0 (median value 1, Inter Quartile range 5) decreased at T4 (median value 0, Inter Quartile range 4), the difference was not significant according to Wilcoxon Signed Rank Test, probably because of small sample size (Fig. 2D).

Hence, to express the cumulative changes in RANK-positive and -negative CTCs with a simple parameter, at individual patient level, we exploited here the parameter ΔAUC that we previously reported for another customized assay^35^.

Briefly, the detected numbers of RANK-positive and -negative CTCs were plotted in relation to time and the difference of the AUC of longitudinal graphs was calculated^35^, obtaining a relative number for each patients. In our cohort (n = 42) the ΔAUC mean value was −113.6 + 352.7 SD, ranged from −1505 to 138, median value 0.

In particular, when over the follow-up we found higher number of RANK-negative CTCs the ΔAUC resulted negative, whilst whenever we found higher number of RANK-positive CTCs, the ΔAUC resulted positive. According to this algorithm, we could also include the 4 out of 42 patients that were CTC-negative at all the considered time-points, attributing them a ΔAUC value of 0.

Hence, ΔAUC value 0 represents the absence of CTCs (n = 4) or an equal number of RANK-positive and RANK-negative CTCs (n = 1). For categorized analyses, patients with positive ΔAUC value (ΔAUC > 0) - expression of RANK-positive CTCs persistence over the follow-up period – were compared with which had negative or 0 ΔAUC (ΔAUC < 0).

**Figure 3:**
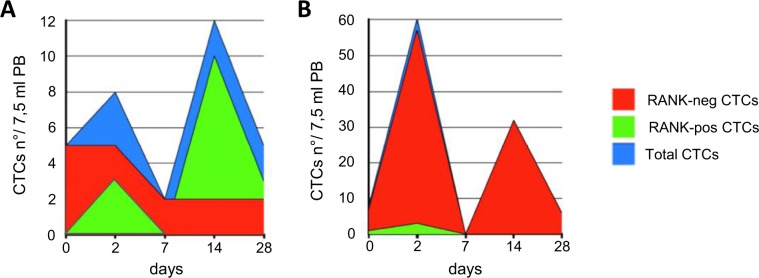
Longitudinal graphs of CTC subsets in two representative patients. (**A**) During Denosumab treatment we registered 2 peaks of CTCs, at 2 and 14 days (blue area). The area under the RANK-negative CTC curve (red) was slighter than RANK-positive CTC curve (green). The ΔAUC value was + 80. The time to first SRE was 50 months; (**B**) in the second case under Denosumab CTCs’ level decreased (blue area). The area under the RANK-negative CTC curve (red) was greater than RANK-positive CTC curve (green). The ΔAUC value was −889. The time to first SRE was 5 months.

The Fig. 3 shows 2 representative cases, with positive (A) and negative ΔAUC (B), respectively.

**Figure 4:**
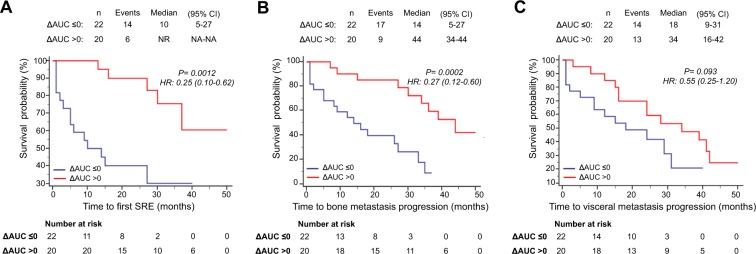
Survival analyses of patients categorized in ΔAUC ≤ 0 or ΔAUC > 0. Kaplan-Meier curves for time to first SRE (**A**), time to bone metastasis progression (**B**) and time to visceral metastasis progression (**C**).

Overall, median follow-up was 38 months (95% CI, 25.4–50.5 months). Median time to first SRE was 10.0 months in ΔAUC ≤ 0 patients (95% CI, 5.0–27.0 months), while it was not reached in ΔAUC positive patients. Moreover, we observed a median time to bone metastasis progression of 14.0 months in ΔAUC ≤ 0 patients (95% CI, 5.0–27.0 months) vs 44 months (95% CI, 34.0–44.0 months) in ΔAUC positive patients, respectively. The univariate analysis showed that a positive ΔAUC was significantly associated with longer time to first SRE (p = 0.0002) and time to bone metastasis progression (p = 0.0012), suggesting that the persistence of RANK-positive CTCs predicted more favorable skeletal outcomes. (Fig. 4A,B). ΔAUC, analyzed as continuous variable, was still associated to better bone outcomes (Table 3).

**Table 3:** Univariate cox regression analysis of ΔAUC and Slope as continuos variables.

Variables	Time to first SRE		Time to bone metastasis progression		Time to visceral metastasis progression	
	p value	HR (95% CI)	p value	HR (95% CI)	p value	HR (95% CI)
ΔAUC	0.0001	0.998 (0.997–0.999)	0.0001	0.998 (0.997–0.999)	0.0054	0.999 (0.998–1.000)
Slope	0.0064	0.983 (0.971–0.995)	0.0108	0.986 (0.975–0.997)	0.1953	0.993 (0.982–1.004)

Abbreviations: SRE, skeletal releated event.

Otherwise, Kaplan Meier survival analyses showed no significant association between ΔAUC value and time to visceral metastasis progression (ΔAUC ≤ 0: median 18 months, 95% CI, 9.0–31.0 months; ΔAUC > 0: median 34 months, 95% CI, 16.0–42.0 months; p = 0.093) (Fig. 4C).

Furthermore, we performed the analysis of the slope of the straight line connecting the pair T0-T1 time-points of RANK-positive and RANK-negative CTCs in 32 patients, whose both time points were available. Patients were dichotomized in positive Slope values for both RANK-negative and RANK-positive CTCs trends (reflecting an early increase respectively in RANK-negative or RANK-positive CTCs counts after first denosumab administration) and negative or 0 slope.

**Figure 5:**
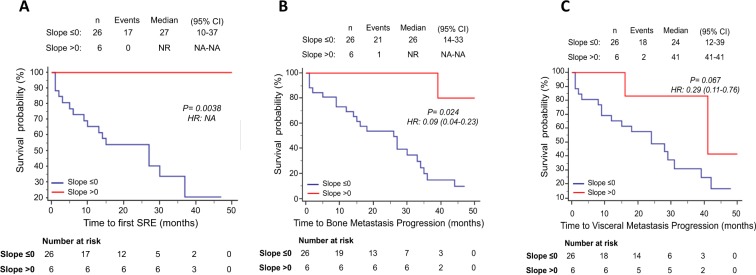
Survival analyses of patients categorized in slope ≤0 or slope >0 for RANK positive CTCs. Kaplan-Meier curves for time to first SRE (**A**), time to bone metastasis progression (**B**) and time to visceral metastasis progression (**C**).

**Figure 6:**
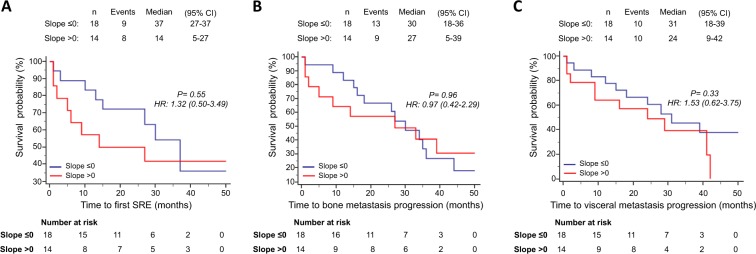
Survival analyses of patients categorized in slope ≤0 or slope >0 for RANK negative CTCs. Kaplan-Meier curves for time to first SRE (**A**), time to bone metastasis progression (**B**) and time to visceral metastasis progression (**C**).

Intriguingly, patients who showed a positive slope for RANK-positive CTCs had no SRE events during all the follow-up time, while slope negative patients reached a median time to first SRE of 27.0 months (95% CI, 10.0–37.0 months) (p = 0.0038) (Fig. 5A). Similarly, median time to bone metastasis progression was significantly shorter (p = 0.0024) in patients with negative slope for RANK-positive CTCs (26.0 months; 95% CI, 14.0–33.0 months) compared to positive slope patients (median time to bone metastasis progression not reached) (Fig. 5B). No statistically significant correlation, on the other hand, were found between positive slope for RANK-positive CTCs and time to visceral metastasis progression (Fig. 5C), as well as between slope for RANK-negative CTCs and any clinical endpoints (Fig. 6).

Slope, analyzed as continuous variable, maintained its ability in predicting prognosis in skeletal endpoints. (Table 3). The prognostic role of clinical variables was explored in univariate analysis finding the presence of visceral metastasis the only parameters, beside ΔAUC and positive slope, correlated with clinical endpoints (Table 4).

**Table 4:** Univariate cox regression analysis of all clinical-pathological variables.

Variables	Time to first SRE		Time to bone metastasis progression		Time to visceral metastasis progression	
	p value	HR (95% CI)	p value	HR (95% CI)	p value	HR (95% CI)
Age	0.825	0.99 (0.96–1.02)	0.853	0.99 (0.97–1.02)	0.461	0.98 (0.96–1.01)
Visceral metastasis	0.071	2.30 (0.93–5.70)	0.100	1.90 (0.87–4.15)	0.011	2.82 (1.27–6.27)
ER	0.476	0.60 (0.23–2.00)	0.757	0.84 (0.29–2.46)	0.598	0.77 (0.29–2.04)
PR	0.614	0.80 (0.33–1.93)	0.143	0.55 (0.25–1.22)	0.853	0.93 (0.42–2.03)
HER2	0.888	1.08 (0.39–2.95)	0.418	1.44 (0.60–3.43)	0.859	0.92 (0.37–2.27)
Bone metastases lytic	0.956	1.03 (0.41–2.57)	0.447	1.36 (0.62–3.01)	0.776	1.12 (0.50–2.50)
Bone metastases sclerotic	0.488	1.47 (0.49–4.41)	0.338	1.61 (0.61–4.29)	0.804	1.15 (0.39–3.38)
Bone metastases mixed	0.150	0.44 (0.15–1.33)	0.117	0.48 (0.19–1.20)	0.601	1.24 (0.56–2.72)
Hormone	0.522	0.75 (0.31–1.80)	0.974	1.01 (0.47–2.19)	0.506	0.77 (0.36–1.65)
Anti-HER2 agents	0.688	1.23 (0.45–3.40)	0.453	0.67 (0.25–1.81)	0.565	0.75 (0.28–1.99)
Chemotherapy	0.750	1.16 (0.47–2.82)	0.919	0.96 (0.43–2.12)	0.164	1.71 (0.81–3.64)
De novo mestastic/Reccurrent	0.326	1.57 (0.64–3.82)	0.422	1.38 (0.63–3.02)	0.301	0.67 (0.31–1.43)

Abbreviation = SREs, Skeletal Related Events; ER, Estrogen Receptor; PR, Progesteron Receptor.

Finally, multivariate cox proportional hazards regression analysis confirmed the ability of positive ΔAUC in identifying patients with significantly better prognosis in terms of skeletal endpoints (Table 5).

**Table 5:** Multivariate cox regression analysis of ΔAUC positive patients.

Variables	Time to first SRE		Time to bone metastasis progression		Time to visceral metastasis progression	
	p value	HR (95% CI)	p value	HR (95% CI)	p value	HR (95% CI)
Visceral metastasis	0.036	2.70 (1.07–6.80)	0.072	2.04 (0.93–4.49)	0.009	2.95 (1.32–6.62)
ΔAUC > 0	0.002	0.20 (0.07–0.55)	0.0006	0.20 (0.08–0.50)	0.775	0.48 (0.21–1.08)

Abbreviation = SREs, Skeletal Related Events.

Moreover, multivariate analyses confirmed that positive slope of RANK-positive CTCs was associated to delay in time to bone metastasis progression, while we could not analyzed the correlation with time to first SRE, since no events occurred (Table 6).

**Table 6:** Multivariate cox regression analysis Slope for RANK positive CTCs.

Variables	Time to first SRE		Time to bone metastasis progression		Time to visceral metastasis progression	
	p value	HR (95% CI)	p value	HR (95% CI)	p value	HR (95% CI)
Visceral metastasis	0.028	3.43 (1.15–10.22)	0.049	2.14 (1.01–5.78)	0.02	3.34 (1.19–9.36)
Slope RANK positive	NA	NA	0.046	0.12 (0.02–0.95)	0.40	0.46 (0.10–2.23)

Abbreviation = SREs, Skeletal Related Events.

## Discussion

In this study, we examined RANK expression in CTCs collected from 42 MBC patients during denosumab therapy by CS system integrated with specific anti-RANK antibody.

Our study shows some limits. The first one depends on the small sample size, which is that proper of a pilot study. Hence, its findings should be generalized with caution, since they warrant further confirmation in larger, prospective studies *ad hoc* designed.

Another limit is related to the method we chose for isolating and characterizing the CTCs. Currently, CS is the only clinically validated FDA-cleared test able to captures and enumerates CTCs, which express EpCAM as well as intracellular cytokeratins (CK). Since CTCs are a heterogeneous population of tumor cells similarly to the primary tumor, this excludes that one test may fit all subset of CTCs, and detractors of the CS method claim for assays that should be “more sensitive”, in order to include not only epithelial CTCs. However, previous studies have demonstrated that the EpCAM expressing CTCs were strongly correlated with poor overall survival, whilst EpCAM-negative CTCs did not show a clinical relevance^38,39^. Since our purpose was to investigate the potential prognostic/predictive value of RANK-positive CTCs, we considered here only EpCAM-positive CTCs. Similarly, to determine the better window of analysis, we chose to limit the time-line of RANK expression on CTCs to the first month of treatment. Since it is known that denosumab activity can be measured *in vivo* within the first week of treatment^37^, we were interested to explore the value of the CTC test for RANK as decision marker at early as possible. Otherwise, the total CTC enumeration has been already reported as prognostic marker in MBC, not only al baseline, before starting a new treatment, but also at the subsequent treatment cycles^40^.

We found at least one RANK-positive CTC in 70% of CTC-positive patients, suggesting that RANK expression may represent a phenotypic (and biologic) property of cancer cells with metastatic behavior. To investigate if this specific CTC subpopulation was associated with clinical outcome in terms of time of bone and/or visceral progression, we correlated the number of RANK-positive CTCs with skeletal and non-skeletal clinical endpoints.

Notably, in our cohort, the baseline number of RANK-positive CTCs lacks association of statistical significance with patients’ outcome. However, when we exploited the algorithm ΔAUC in survival analyses showed that the persistence of RANK-positive CTCs (positive ΔAUC), during denosumab administration, was associated with longer time to first SRE and time to bone metastasis progression, but not with delay in time to visceral metastasis progression. Despite this seems to be counterintuitive – RANK expression in primary tumor is unfavorable prognostic marker at bone level^17,18^ - this data is the main finding of our study, since it correlates directly the maintenance of RANK expression with the effectiveness of its specific target drug, i.e. denosumab.

This data also suggests that RANK positive CTCs could play a potential surrogate prognostic/predictive role in the specific context of bone metastases. Indeed, although it could be difficult to respect in clinical routine the time-line of multiple blood tests used here, the analysis of the Slope confirmed the correlation between RANK-positive CTCs and increased time to first SRE and time to bone metastasis progression. This suggests also that more was the increase of RANK-positive CTCs at the 2° day of treatment, more efficacious was the therapy. The translational relevance of this “brief test” is evident.

The finding of “brief test” appears to be in contrast with previously reported data, since a decrease in CTCs counts during oncological therapies is generally associated with favorable outcomes and treatment responsiveness, while the increase in CTCs numbers is commonly associated to the release of CTCs from progressive disease^41^.

Otherwise, Vetter and colleagues^42^ recently reported in a small size sample (n = 20) that some breast cancer patients turned negative during denosumab treatment.

Main methodological differences between the two studies prevent a direct comparison of the data, namely the criteria for defining a cell as CTC - EpCAM + CD45- in Vetter study vs. EpCAM + CK + DAPI + CD45- in our study-, and the procedure for CTC enumeration - manual vs. automated procedure, respectively. Moreover, Vetter and colleagues^42^ did not describe the time-line schedule of blood draws subsequent the baseline one, hence we cannot compare time-matched CTC enumeration.

We interpret the rise in RANK-positive CTC number at T1 because of CTC mobilization from bone microenvironment in bloodstream. Indeed, previous evidences have described CTC mobilization from the bone marrow after treatment with hematopoietic stem cells (HSCs) mobilizing agents such as granulocyte colony-stimulating factor (G-CSF) and a CXCR4 inhibitor. These mobilizing drugs enhanced cancer cell sensitivity to chemotherapy promoting them from dormancy into the cell cycle^43^. From this perspective, the raise of RANK-positive CTCs in patients who have obtained the higher skeletal benefit, may reflect the ability of denosumab to effectively disrupt RANKL/RANK signaling leading to reduction of cancer cell retention in bone and, hence, a paradoxical release of RANK-positive CTCs in bloodstream.

Our results highlighted that metastatic breast cancer cells exhibited different phenotypical profiles and this heterogeneity influenced therapy response. The high heterogeneity of breast tumor could be one explanation for the conflicting results of the two phase III trials ABCSG-18 and D-CARE^21,22^, underlining the need to predictively select those patients could benefit from therapy. In this regard, it is conceivable that may exist a subgroup of patients that could more benefit from denosumab treatment. On the bases of our results, we speculate that RANK expression on tumor cells may contribute to identify this subset of patients.

In conclusion, we demonstrated, for the first time, the presence of RANK-positive CTCs in bloodstream of MBC patients and that *in vivo* the effectiveness of Denosumab depends on the expression of RANK. Our findings offer a rationale to design future prospective clinical trials for changing vs continuing initial therapy, utilizing the “brief” CTC test for RANK as predictive marker. More in general, we provided evidences that CTCs allow monitoring serial changes of cancer biology, revealing the spatial and temporal heterogeneity of individual tumor undergone a specific treatment.

## Methods

### Study design

The study was conducted at Campus Bio-Medico University of Rome, authorized and approved by the Ethics Committee and our Institutional Review Board (Prot: 40/15 OSS). All procedures were performed in accordance with the Declaration of Helsinki. Informed consent was obtained from all patients prior to study entry.

We planned to perform a pilot study and our estimation of patient number was only rough and based on published findings of CTC-positive patients in metastatic breast cancer, since we did not know the prevalence of RANK-positive CTCs, as determined with our integrated assay.

Hence, we planned to recruit at least 30 evaluable patients according to Browne^44^, which estimated this sample size as adequate for exploratory analysis.

Forty-two MBC patients with bone metastases candidates to receive denosumab concomitantly to oncological treatments were recruited into the study between April 2011 and November 2015.

All the patients received the recommended dose of 120 mg denosumab, as a single subcutaneous injection once every 4 weeks - into the thigh, abdomen or upper arm, for prevention of skeletal related events in adults with bone metastases from solid tumors.

Peripheral blood samples were collected at baseline and at day 2, 7, 14, 28 after the first denosumab administration (T0, T1, T2, T3, and T4). Medical records were reviewed and clinical characteristics were collected retrospectively including age, tumor intrinsic subtype, oncological treatments, Eastern Cooperative Oncology Group (ECOG) performance status, presence of visceral metastases, time to first SRE, time to bone metastasis progression and time to visceral metastasis progression. Time to first SRE is defined as the time between the date of the start of denosumab and the date of first SRE onset including pathologic fracture, spinal cord compression, necessity for radiation to bone (for pain or impending fracture) or surgery to bone. Time to bone metastasis progression is defined as the date of the start of denosumab and the date of bone radiological progression (according RECIST criteria) and/or SRE. Time to visceral metastasis progression is defined as the date of the start of denosumab and the date of visceral radiological progression (according RECIST criteria).

### Total and RANK-positive CTC count

We used the CellSearch System (Menarini Silicon Biosystems, LLC), to enumerate CTCs in peripheral blood, according to manufacturer’s instructions and users’ guidelines^25,45^. Briefly, an event is classified as a CTC when its morphological features are consistent with that of a cell and it exhibits the phenotype EpCAM + , cytokeratin 8, 18, 19 + (CK + ), DAPI + and CD45-.

**Figure 7:**
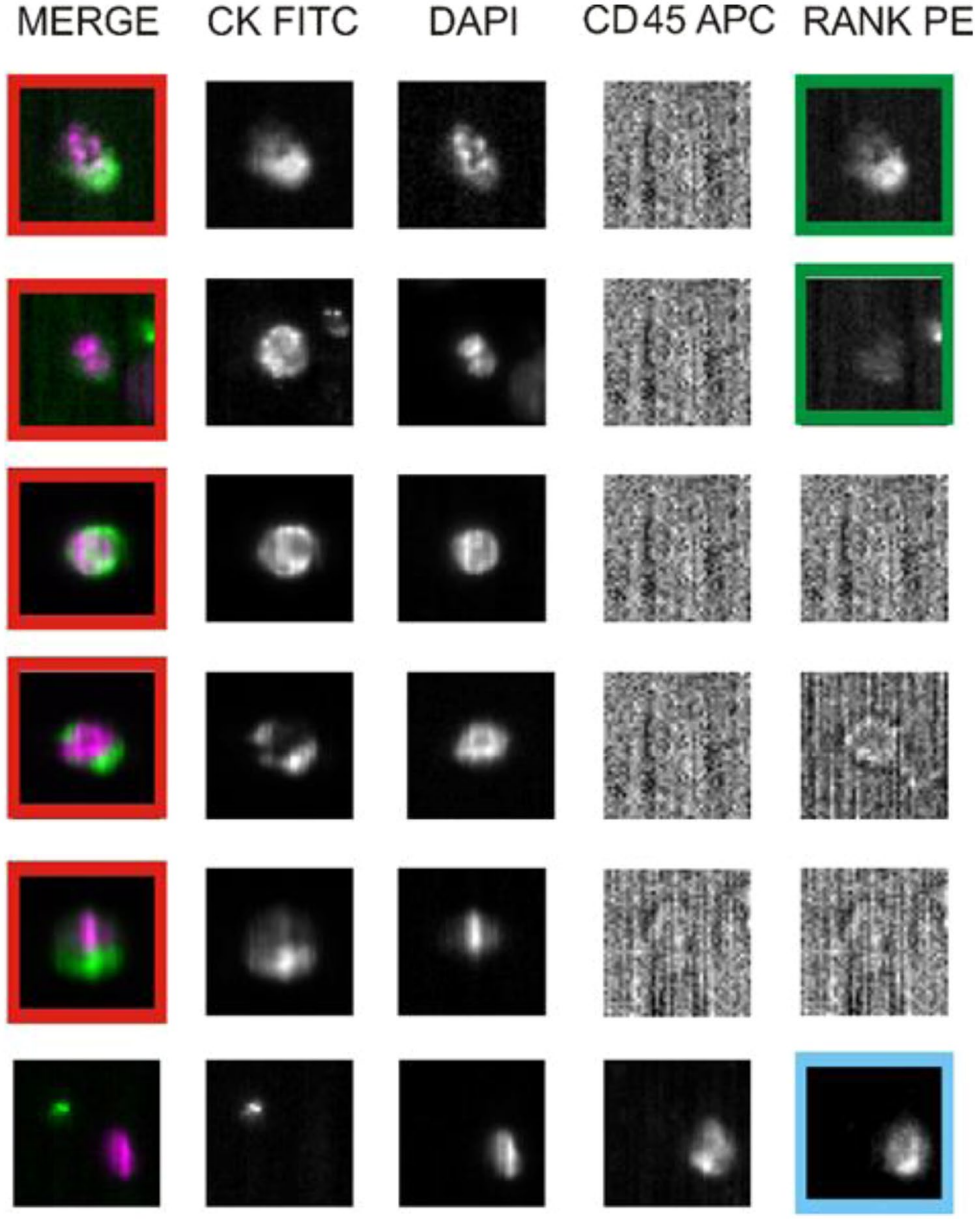
RANK immunostaining of CTCs *ex vivo*. Analysis of five rare cells and one leukocyte in a blood sample of a breast cancer patient using an Analyzer II device. Horizontally, the photos show the same cell stained for the combination (DAPI/CK FITC) of CK (green) and DAPI (violet); CK FITC only; DAPI only; CD45 APC only; and RANK PE only. The red squares indicate positively stained cells: based on RANK staining profile (sufficient signal relative to background) we classified the first two cells as RANK-positive CTCs (green squares). Based on CD45 expression, the bottom photos show a leukocyte that express RANK (light blue square), and serve as internal fluorescence control.

RANK-positive CTCs were identified as previously reported for another customized test^35^ by integrating the standard assay with a specific mAb for detecting RANK expression (Fig. 7). Result has been expressed as the total number of CTCs and RANK-positive CTCs per 7.5 ml of blood at each time-points (T0, T1, T2, T3, and T4).

### Statistical analysis

We developed a companion algorithm (ΔAUC) to express the difference between RANK-positive and RANK-negative CTC concentration-time area (AUC), as calculated according to the following formula, we previously applied to another customized CTC test^35^:

$$\Delta {\rm{AUC}}={\rm{RANK}} \mbox{-} {\rm{positive}}\,{\rm{CTC}}\,{\rm{AUC}}-{\rm{RANK}} \mbox{-} {\rm{negative}}{\rm{CTC}}\,{\rm{AUC}}$$

Kaplan-Meier method and Mantel-Haenszel log-rank test were performed to compare survival among groups in terms of median time to first SRE, median time to bone metastasis progression, and median time to visceral metastasis progression. All these clinical variables were estimated by univariate and multivariate cox regression when ΔAUC and slope were analyzed as continuous variables. Variables to be included, beside ΔAUC and slope, in multivariate analysis were selected according the levels of significance in cox regression univariate analysis (threshold set at p = 0.1). P-values < 0.05 were considered significant. All statistical analyses were performed using SPSS software (version 19.00, SPSS, Chicago)
